# Tree aboveground carbon storage correlates with environmental gradients and functional diversity in a tropical forest

**DOI:** 10.1038/srep25304

**Published:** 2016-06-09

**Authors:** Yong Shen, Shixiao Yu, Juyu Lian, Hao Shen, Honglin Cao, Huanping Lu, Wanhui Ye

**Affiliations:** 1 Department of Ecology, School of Life Sciences/State Key Laboratory of Biocontrol, Sun Yat-sen University, Guangzhou 510275, P.R. China; 2 Key Laboratory of Vegetation Restoration and Management of Degraded Ecosystems, South China Botanical Garden, Chinese Academy of Sciences, Guangzhou 510650, P.R. China; 3 Ecological Meteorological Center of Guangdong Province, Guangzhou 510640, P.R. China

## Abstract

Tropical forests play a disproportionately important role in the global carbon (C) cycle, but it remains unclear how local environments and functional diversity regulate tree aboveground C storage. We examined how three components (environments, functional dominance and diversity) affected C storage in Dinghushan 20-ha plot in China. There was large fine-scale variation in C storage. The three components significantly contributed to regulate C storage, but dominance and diversity of traits were associated with C storage in different directions. Structural equation models (SEMs) of dominance and diversity explained 34% and 32% of variation in C storage. Environments explained 26–44% of variation in dominance and diversity. Similar proportions of variation in C storage were explained by dominance and diversity in regression models, they were improved after adding environments. Diversity of maximum diameter was the best predictor of C storage. Complementarity and selection effects contributed to C storage simultaneously, and had similar importance. The SEMs disengaged the complex relationships among the three components and C storage, and established a framework to show the direct and indirect effects (via dominance and diversity) of local environments on C storage. We concluded that local environments are important for regulating functional diversity and C storage.

Understanding how biodiversity affects ecosystem functioning is a fundamental aim in ecology^1^,^2^,^3^. There has been increasing interest in the relationship between functional diversity and carbon (C) storage in the context of global climatic change over the past few decades^4^, especially in natural forest ecosystems^5^,^6^.

Studies have linked plant functional traits to C storage^5^,^6^, since tree species with different characteristics have distinguishing abilities to capture, store, and release carbon^4^. Some studies have shown that the functional diversity index has the potential to quantify these functional characteristics^5^,^6^, because it reflects the value, range and relative abundance of the functional traits of species in a community^7^. Other studies have presented several key functional traits that relate to some important variation in ecological strategy among species, and the underlying adaptive trade-offs between the traits^8^,^9^,^10^. For instance, specific leaf area (SLA) measures light-intercepting leaf area per dry mass invested^11^,^12^, and leaf dry matter content (LDMC) reflects the amount of assimilatory tissue versus structural compounds found in a leaf^13^. Wood density (WD) represents biomass invested per unit volume^9^,^10^, while maximum tree height is associated with light competition ability among species^5^. Thus, a range of species characteristics may be related to the potential ability to determine C storage^5^,^14^.

While the relationship between functional diversity and C storage has been evaluated, inconsistent results have been observed in different ecosystems and it remains unclear how functional dominance and functional diversity regulate C storage. Ruiz-Jaen & Potvin^5^ found that results were very different between a plantation forest and natural forest^5^. Species richness explained most of the variation in C storage in the plantation forest, but that was not the case for the natural forest, where explanation of the variation in C storage depended on functional dominance (dominant species or traits determined C storage). Conti & Diaz^4^ showed that functional dominance of maximum tree height was negatively associated with C storage, while functional divergence of WD was significantly positively correlated with C storage in semi-arid forest ecosystems^4^. In contrast, other studies have demonstrated that both taxonomic diversity and functional dominance were positively correlated with C storage^6^. These inconsistencies imply that mechanisms that drive relationships between functional dominance, functional diversity and C storage differ greatly in different ecosystems. A significant dominance - C storage relationship may result from selection effects, meaning that dominant species or traits are the main drivers of ecosystem functioning. A positive diversity - C storage relationship can be driven by complementarity effects, in which diverse traits can better utilize limited resources and lead to high C storage in an ecosystem^6^.

A number of significant research questions remain in relation to these inconsistencies. First, studies focused on C storage distribution across forest types or regions at large scale^4^,^5^,^6^, but fewer studies have tested the fine-scale variation in C storage in tropical forests^28^,^29^. Also, little is known about the internal variation in C storage, which could add uncertainty to empirical relationships between environmental variables, functional diversity and aboveground C storage^6^. Second, studies have linked biodiversity to C storage using experimental plantations^15^,^16^, since they allow for testing the mechanisms (i.e. complementarity or selection effects) responsible for the linkage separately, but plantations do not contain the natural variability that occurs in natural forests^5^. As a result, much less is known about the mechanisms involved functional diversity - C storage relationships in more complex natural tropical forests^5^,^6^. Furthermore, we do not know if results found in mixed-species plantations can be extrapolated to predict what is found in natural forests^5^. More observational studies are needed to fill this gap. Third, studies have also found that use of a single multivariate functional diversity index can mask some key relationship between diversity and ecosystem functioning^17^, because different individual functional traits could be related to opposing niche axes, and are associated with different ecosystem functioning^18^,^19^. For example, tree height and leaf size are linked to plant stature, but SLA, LDMC and chlorophyll concentration are related to leaf traits and resource acquisition. Thus, the use of single trait diversity to investigate fine-scale variation in C storage in natural tropical forests could help understand the diversity - productivity relationships.

In addition, environmental factors have important effects on the relationship between diversity and ecosystem functioning, but studies at large scales may also mask some important connections if local environmental factors are not taken into account^6^,^20^,^21^,^22^. Cavanaugh *et al*^6^ investigated the influence of large-scale environmental gradients and functional diversity on C storage^6^, but they did not find any significant relationships between environmental factors and C storage, they suggest that these relationships can be obscured by limitations in the accuracy of estimates of C storage and environmental data. This is particularly pertinent in environmental data at larger scale, which are dependent on the density of climate stations near study sites and the quality of the interpolation methods used. However, Ma *et al*^22^ found that large-scale environmental gradients had significant effects in regulating the relationship between species diversity of grasses and aboveground productivity^22^. Previous studies also demonstrated that fine-scale heterogeneity of habitat can result in largely differential availability of water and nutrients in an ecosystem^23^, and these directly relate to plant performance and ecosystem processes^24^,^25^,^26^. McEwan *et al*^27^ and Xu *et al*^28^ found that local topographic factors, such as convexity and slope, can be predictors of aboveground C storage in tropical forests^27^,^28^, and Lin *et al*^29^ also found that aboveground biomass correlated with fine-scale habitats^29^. These findings imply that local environment may play a key role in determining the relationships between functional dominance, functional diversity and aboveground C storage. Therefore, local environments should be emphasized in seeking to characterize diversity - productivity relationships.

In the present study, we examined how fine-scale environmental factors, functional dominance and functional diversity influenced aboveground C storage in trees in the Dinghushan (DHS) 20-ha Forest Dynamics Plot in Southern China. We also evaluated the relative importance of fine-scale environmental factors, functional dominance and diversity in regulating C storage distribution. We addressed the following three hypotheses: (1) there is large fine-scale variation in aboveground C storage in the DHS tropical forest, as a result of largely heterogeneous habitats in this study site; (2) functional dominance and functional diversity have significant effects on C storage of quadrats, reflecting the relative importance of complementarity effects and selection effects under different local habitats; and (3) fine-scale environmental factors play key roles in determining aboveground C storage, functional dominance and functional diversity in this tropical forest, since heterogeneous environments can result in largely differential water and nutrient availability, even at small scales, and then affects plant performance. However, as biotic factors, functional dominance and functional diversity may be more important in regulating aboveground C storage than fine-scale environmental factors, because plant performance could affect C storage directly in a tropical forest.

## Results

### Univariate correlations among environmental factors, functional dominance, functional diversity and C storage

**Figure 1:**
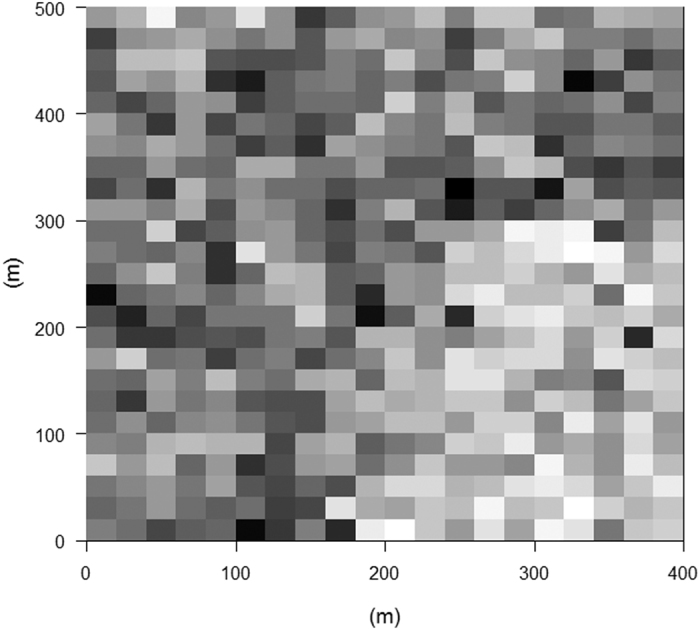
C storage distribution at the scale of 20 m × 20 m in DHS plot. C storage from 10.8 (white) to 240.7 (black) Mg C ha^−1^.

The mean C storage was 103.19 Mg C ha^−1^ in the DHS plot, and this value was very close to the results reported in Tang *et al*^30^. This result supported the validity of the C storage estimation method. Tang *et al*^30^ used different methods (a local model) to estimate aboveground biomass and measured C contents of dominant species to calculate C storage in a 1-ha plot in this site, and found that C storage was 106.80, 109.85 and 89.75 Mg C ha^−1^ in 1992, 1994 and 1999, respectively. However, the variation in C storage across different quadrats was very large (Fig. 1), ranging from 10.8–240.7 Mg C ha^−1^, which was consistent with our first hypothesis. The distribution of C storage showed a significant spatial pattern, it was much lower in the quadrats of southeast corner, and higher in other quadrats (Fig. 1). Fine-scale environmental factors, functional dominance and functional diversity were highly correlated with C storage (Table 1), and this was in line with the expectations of our second and third hypotheses. All relationships were significant except for functional diversity of WD and C storage. However, both positive and negative relationships were detected for functional dominance and diversity of different traits. Multivariate functional diversity was negatively associated with C storage. Functional dominance and functional diversity of each trait was also highly positively correlated (Fig. S1), except for LDMC (negative), and this was consistent with the relationship between functional dominance, functional diversity and C storage of each trait (Table 1). Interestingly, high convexity had positive effects on C storage, but in the higher soil fertility (high PC1) quadrats, C storage was relatively lower (Table 1). Our results also showed that functional dominance and diversity were strongly influenced by terrain convexity and soil fertility (Table 2), which supported our third hypothesis. Nevertheless, the diversity of WD was not significantly associated with convexity and soil PC1.

### Structural equation models relating environmental factors, functional dominance, functional diversity and C storage

**Figure 2:**
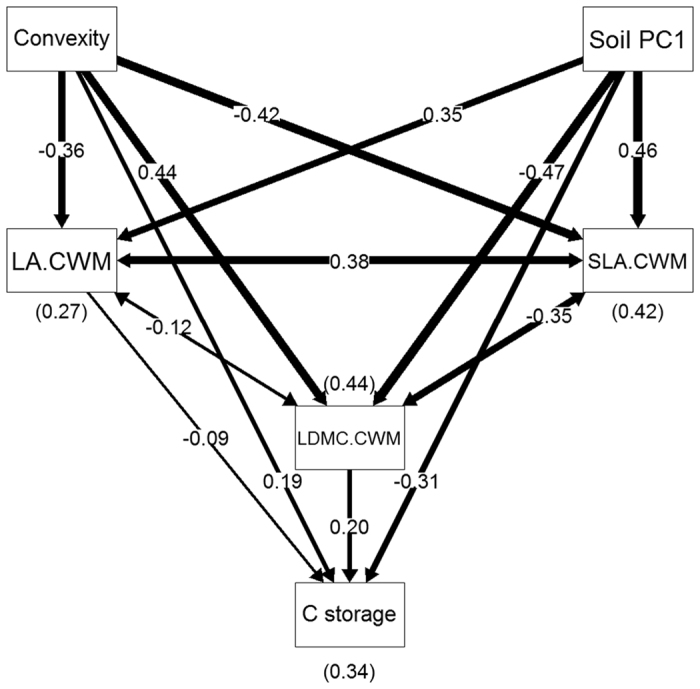
Structural equation model relating C storage, functional dominance of leaf traits and fine-scale environmental factors in DHS plot. Single headed arrows indicate directional relationships, while double headed arrows indicate covariances. Thicker lines correspond to stronger relationships, and numbers in brackets are R^2^ values. See Table 1 for abbreviations.

**Figure 3:**
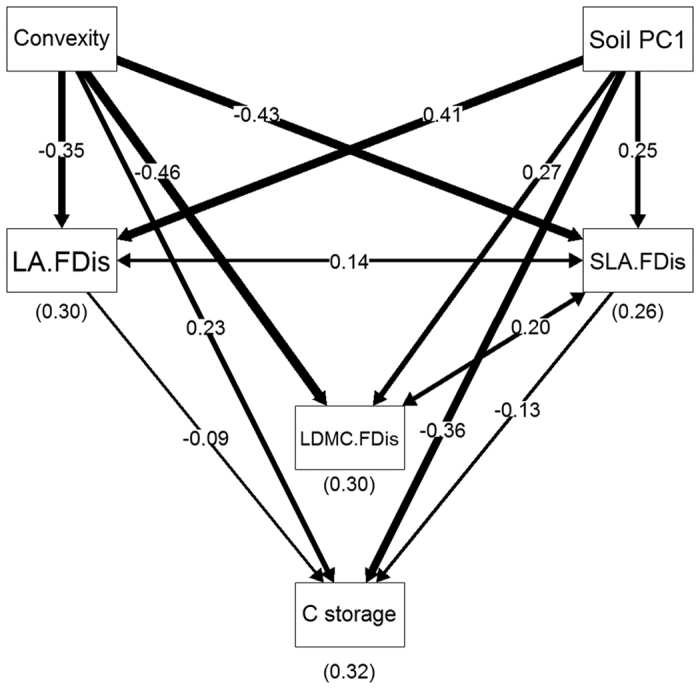
Structural equation model relating C storage, functional dispersion of leaf traits and fine-scale environmental factors in DHS plot. Single headed arrows indicate directional relationships, while double headed arrows indicate covariances. Thicker lines correspond to stronger relationships, and numbers in brackets are R^2^ values. See Table 1 for abbreviations.

Our structural equation models (SEMs) also indicate that there were strong relationships among fine-scale environmental factors, functional dominance, functional diversity and C storage (Figs 2 and 3), in accordance with our second and third hypotheses. In the SEM for functional dominance (χ^2^ = 0.23, N = 500, d.f. = 3, P = 0.97, AIC = 36.23), convexity, soil PC1 and functional dominance of the three leaf traits explained a total of 34% of the variation in C storage (Fig. 2), but the model did not detect a direct effect of SLA on C storage. Convexity and soil PC1 had important effects on functional dominance of leaf traits, particularly on functional dominance of LDMC and SLA (R^2^ = 0.44 and 0.42, respectively). For the SEM of functional diversity (χ^2^ = 2.69, N = 500, d.f. = 3, P = 0.44, AIC = 38.69), a similar proportion of variation in C storage can be explained by fine-scale environmental factors and functional diversity of leaf traits (R^2^ = 0.32) (Fig. 3), but the insignificant association between LDMC and C storage was removed in this final model. Nevertheless, compared with the functional dominance model, convexity and soil PC1 had weaker relationships with functional diversity of leaf traits, since R^2^ ranged from 0.26–0.30.

### The best predictor of C storage

**Figure 4:**
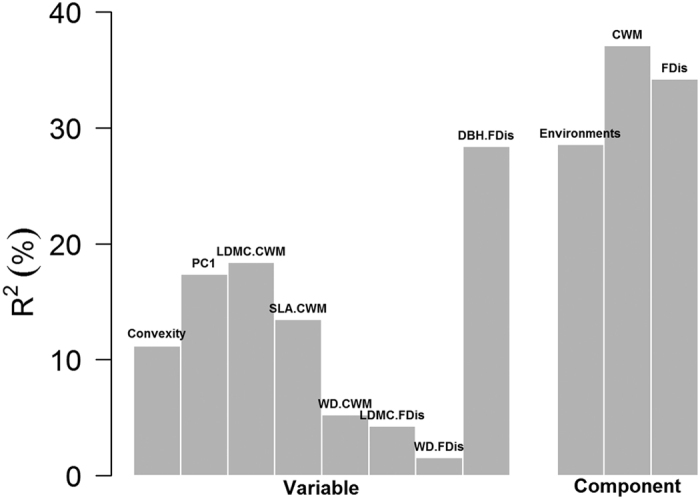
Relative importance of each regressor and component in the final stepwise multiple regression model for all variables in the DHS plot. See Table 1 for abbreviations.

We used stepwise multiple regression models to estimate the relationships between C storage and the different components of the variables. The R^2^ of final models ranged from 0.30–0.42 (Table 3). Functional dominance and diversity explained similar proportions of variation in C storage in the regression models. The models were improved slightly when convexity and soil PC1 were taken into account (R^2^ increased). The model that included all variables explained the largest proportion of variation in C storage (R^2^ = 0.42). When we decomposed the R^2^ of the final model, we found that functional diversity of DBH (diameter at breast height, 1.3 m) was the best predictor of C storage (Fig. 4). We also learned that functional dominance contributed the largest proportion of R^2^ to the final model, and that functional dominance and functional diversity contributed more to R^2^ than environments, which was consistent with the prediction of our third hypothesis.

## Discussion

Tropical forests are the biologically richest ecosystems in the world, and play a disproportionately important role in the global C budget^6^,^31^. In our study, we found significant effects of local environments, functional dominance and functional diversity on fine-scale variation in C storage in a tropical forest, which helped to unravel the complex relationships among the three components and C storage, and provided new insights into the diversity - productivity relationships.

We are confident that the estimation method of aboveground C storage in trees used in Chave *et al*^32^, Ruiz-Jaen & Potvin^5^ and Cavanaugh *et al*^6^ is appropriate for our study site^5^,^6^,^32^, because our C storage values were very close to that of these other studies, which used different estimation methods at the same site^30^. We also found that functional dominance was strongly associated with C storage, because all relationships between functional dominance and C storage were significant in this study (Table 1), a result that supported our second hypothesis. Functional dominance of LDMC, WD and DBH were significantly positively correlated with C storage, and functional dominance of LA and SLA were significantly negatively associated with C storage, which was consistent with previous study^5^. However, this result was not consistent with Conti & Diaz^4^, who did not find functional dominance and diversity of leaf traits as predictors of C storage. These significant relationships support the selection effect hypothesis, which proposes that these dominant species or functional traits (higher or lower values of different traits) are the most important factors for driving ecosystem functioning^6^,^33^. Nevertheless, the findings of Finegan *et al*^20^ are very different from ours^20^, they found that functional dominance of SLA was significantly positively correlated with aboveground biomass, and that functional dominance of WD had negative relationship with aboveground biomass. They suggest that the high CWM of SLA denote a community dominated by fast-growing species that maximize resource acquisition and are expected to be associated with high productivity, but high CWM of LDMC and WD indicate a community dominated by conservative species (species with lower resources acquisition ability and growth rates, but higher ability to tolerate shade and other stresses) and are expected to be associated with low productivity. However, this may not be the case for an old-growth forest, such as the DHS plot^23^, since old-growth forests are usually not dominated by fast-growing acquisitive species (i.e. pioneer species with high LA and SLA). Conservative species with larger DBH, higher WD and LDMC, lower LA and SLA can accumulate more C in old-growth forests.

Our study also detected significant relationships between functional diversity and C storage, except for WD (Table 1), which was consistent with our second hypothesis, but was not consistent with Cavanaugh *et al*^6^, since they did not find a significant relationship between functional diversity and C storage^6^. Interestingly, functional diversity of different traits had opposing effects on C storage. The positive relationships between LDMC and C storage, as well as DBH and C storage support the complementarity-effect hypothesis, which suggests that diverse species or functional traits can utilize limited resources effectively and improve total ecosystem functioning^1^,^6^, while other traits (LA, SLA and multivariate) do not support this hypothesis. We suggest that the opposing relationships between functional diversity and C storage may be due to the fact that different functional traits are often associated with different niche axes and ecological processes^17^, which means that the diversity of some traits may promote species in the capture of C. However, diversity of some other traits may reduce the C accumulation of species. For instance, in this study, diverse LA and SLA reduced C accumulation in this forest, which may be due to the fact that LA correlates with respiration and transpiration costs^34^, and SLA correlates with leaf area per dry mass invested and metabolic rates^12^. Therefore, functional diversity cannot be represented by a single multivariate index, and a diversity of individual traits should be included to avoid masking some key relationships between diversity and ecosystem functioning^17^,^19^.

One of the most important findings in this study was that fine-scale environmental factors apparently influence C storage (Table 1, Figs 2 and 3). Environmental variables at large scale were not associated with C storage directly, possibly due to limitations in the accuracy of the environmental data^6^. Whereas, local environmental variables were estimated accurately in our study, and we found some important relationships between environments and C storage, this was due to the fact that fine-scale heterogeneity of habitat can result in differential water and nutrient availability, and this is directly related to plant performance^23^,^24^. Compared with trees in low convexity quadrats, canopy and sub-canopy trees in high convexity quadrats may fully expose the upper canopy layer and capture more light^35^,^36^, which supports high C storage (Table 1, Figs 2 and 3). This mechanism is similar to the results from other studies showing that higher maximum tree height was correlated with higher C storage^4^,^5^,^6^. A few studies detected the relationships between fine-scale topographic factors and aboveground biomass or C storage^27^,^28^,^29^. They also found that forest biomass increased with increasing convexity, high biomass occurred in the quadrats with large convexity located in the relatively flat ridges. However, soil fertility (PC1) negatively affected on C storage (Table 1, Figs 2 and 3). We suggest that high soil fertility corresponds to high diversity of species or traits, particularly in leaf traits (Table 2, Fig. 3), and diverse species characteristics can better utilize limited resources^6^. However, in these quadrats, more space and resources could be occupied by non-canopy and non-dominant trees, and this may result in a low C storage.

Interestingly, our SEMs explicitly showed complex paths from fine-scale environmental factors to functional dominance and diversity of leaf traits, and then to C storage (Figs 2 and 3), which provided supporting evidence for the third hypothesis. We detected direct and indirect effects of environmental factors on C storage. In this analysis, 26–44% of the variation in functional dominance and diversity of leaf traits was explained by convexity and soil fertility (Figs 2 and 3). The two SEMs explained a similar proportion of variation in C storage (R^2^ = 0.34 and 0.32, respectively), which indicates that functional diversity of leaf traits was nearly as important as dominance^6^. Functional traits reflect species strategies in response to environmental variation, thereby strong relationships can be found between traits and environments, as some other studies have reported^37^,^38^. Moreover, there is evidence that a suite of coordinate leaf traits (such as SLA and LDMC) drives a trade-off between acquisition and conservation. This means that a suite of species characteristics promotes fast C acquisition and fast decomposition, while another set of characteristics promotes conservation of resources and slow decomposition^4^,^11^,^39^.

Finally, stepwise multiple regression models were performed to investigate the relative importance of different variable groups. Both functional dominance and diversity were important in predicting C storage in our study. Similar variation was explained by both components (Table 3), which demonstrates that both complementarity effects and selection effects contributed to C storage in the DHS plot. This is consistent with the conclusion of Cavanaugh *et al*^6^. Our results also showed that fine-scale environmental factors apparently contributed to predictions of C storage in this forest, since the R^2^ of the regression models increased when convexity and soil PC1 were taken into account (Table 3). Functional diversity of max DBH was the best predictor of C storage in our study (Fig. 4), indicating that diverse max DBH was the most important factor in C accumulation. This may be due to the fact that max DBH relates to the potential height a species can reach, and is associates with the light capture strategy of the species. A diverse light capture strategy can better utilize the limited light in a tropical forest and supports high C storage, which is consistent with complementarity effects^6^,^40^. Environmental factors, functional dominance and functional diversity play remarkable roles in regulating ecosystem functioning and vary with different ecosystems^4^,^5^,^6^. However, functional dominance and diversity can explain larger proportion of variation in C storage in this tropical forest, a finding that conformed to our expectation in the third hypothesis.

In this study, we sought to understand the complex relationships among fine-scale environmental factors, functional dominance, functional diversity and C storage. Few previous studies have examined these relationships in natural species-rich tropical forests^5^, and our findings provide several new insights into C storage in tropical forests. Functional diversity should be separated into each trait when related to C storage, and local environmental factors should be taken into account in case some key relationships are masked. The prediction of C storage can vary with different ecosystems, and complementarity effects and selection effects can contribute to ecosystem functioning simultaneously, even within the same ecosystem. However, in order to simplify the models and to reduce multicollinearity, our study did not include other environmental variables and functional traits, e.g. altitude, slope and leaf N and P concentration, because functional traits are highly inter-correlated^12^,^37^. We only chose the most important variables based on our previous studies^23^,^41^, which may reduce the predictive ability for C storage. This study focused on the effects of diversity on C storage and did not consider the differences in tree density caused by temporal and occasional natural disturbances (i.e. gap) in this long-term protected site that may contribute to the spatial variability of C storage, and should be taken into account in further studies. Overall, our study has established a framework to understand the interactions of the environment, tree attributes, and ecosystem functioning in a species-rich ecosystem, aiming to improve the conservation and management of tropical forests.

## Methods

### Study site

This study was conducted in a 20-ha (400 m × 500 m) DHS Forest Dynamics Plot in the center of the Dinghushan Nature Reserve (1155-ha) in Guangdong Province, southern China (23° 09′21″–23°11′30″N, 112°30′39″–112°33′41″E). This forest has been conserved for over 400 years. Mean annual precipitation is 1927 mm, and mean annual temperature and humidity are 20.9 °C and 85%, respectively. The DHS plot was established in 2005 following the standards of the Center for Tropical Forest Science (CTFS, http://www.ctfs.si.edu/). The elevation of the plot ranges from 230 m to 470 m. All stems within the plot with DBH ≥ 1 cm were measured, mapped, tagged and identified to species in 2005, 195 species and 71,458 individuals were recorded. The DHS plot was resurveyed in 2010 and 178 species and 61,125 individuals were recorded^42^.

### Fine-scale environmental variables

The 20-ha Forest Dynamics Plot was divided into 500 quadrats (each 20 × 20 m). The small quadrat size can control for the effect of habitat heterogeneity, and can be estimated relatively accurately environmental variables. It also reflects the scale of individual interaction, as neighbor effects were detected in radius of <20 m^5^. To simplify the models, we chose terrain convexity for the topographical index (Table S1). We related terrain convexity to plant performance in one of our previous studies^23^. In 2005, the altitude of the four corners in each quadrat was measured using an electronic station, and the altitude of each quadrat was calculated as the average altitude of its four corners. The terrain convexity of each quadrat was calculated as the altitude of the focal quadrat minus the average altitude of the eight quadrats around the focal quadrat. The convexity of each edge quadrat was calculated as the altitude of the center point minus the mean altitude of its four corners^43^. High convexity may indicate a hilltop, while low convexity may indicate bottomlands or a local hollow^23^.

Relating forest ecosystem functions to edaphic variables can be challenging since vegetation can also affect soils^44^,^45^. We were interested in the effects of soil variables on C storage rather than possible vegetation-caused changes in soil properties, hence we emphasized how soil variables regulated C storage in this study. We used short-term soil variables to relate to long-term C storage because measuring soil properties in different years might not affect their values much in a specific site with relatively stable climate and environment^21^,^44^,^46^. We measured soil properties in a 30 m grid of points in the DHS plot. Two additional sample points at 2, 5, or 15 m in a random compass direction from the grid were added, and a total of 710 samples were collected^23^. At each point, 500 g topsoil samples (0–10 cm) were collected and were analyzed for nine soil properties: total and available N, P, K (mg g^−1^), organic matter (mg g^−1^), water content (%), and pH (Table S1). Soil properties of each 20 × 20 m quadrat were calculated using ordinary Kriging methods^47^. Soil variables were strongly correlated with each other. In order to simplify the models, and to eliminate the effects of multicollinearity, we computed principal components (PCA) from the nine soil variables and used only the first components (PC1), which can explain 64.9% of the total variance in soils variables. Soil PC1 was associated with high soil fertility (Table S2). Variations in topographic and soil variables represented the fine-scale environmental gradients in our study.

### Functional traits and diversity

We measured leaf area (LA, cm^2^), SLA (cm^2^ g^−1^), LDMC (g g^−1^), WD (g cm^−3^) and maximum DBH (cm) as functional traits for the most common 92 species in the plot (Table S1). These 92 species make up 95.5% of the cumulative community basal area in the DHS plot, and are the most important species in determining ecosystem function. We collected relatively young but fully expanded canopy leaves from the six largest and six smallest individuals in the DHS plot^48^. LA was evaluated using a scanner and image processing software (ImageJ, 1.43 u). Leaves were weighed for fresh mass after scanning, and then dried in an oven at 60 °C for at least 72 h to determined dry mass weight. SLA was calculated as the ratio of leaf area to dry mass, and LDMC was determined by dividing leaf dry mass by fresh mass. Maximum DBH was determined by the individual with maximum DBH for each species found in our study site. Maximum DBH can be used as a functional trait since it indicates maximum diameter a species can reach at maturity^5^, and can serve as a proxy for potential height of a species, which is considered an important indicator of the light capture strategy^6^,^40^.

WD was calculated as the ratio of wood dry mass to its fresh volume. We collected wood samples outside the plot using an increment borer, at least six individuals were sampled for each species. Tree cores with 1 cm diameter were collected on the main stem of an individual for trees larger than 6 cm DBH. For shrubs and small trees, we cut 10 cm long and 1 cm diameter stem segments from terminal branches. We used water displacement methods to assess the volume of wood samples, and then dried samples at 60 °C for at least 96 h to determine dry mass^23^.

Community weighted mean (CWM) trait values were calculated as the mean trait value weighted by species abundance in the community^49^. We used species abundance as a weighting factor, but not biomass or basal area, since our analysis tried to link functional dominance to C storage, biomass and basal area are highly correlated with C storage. Calculation of C storage and functional dominance would be dependent on each other if we used biomass or basal area as a weighting factor, and this would potentially mask the real relationships between them. This method has been used in many other studies^4^,^6^,^25^,^49^. We estimated CWM for each functional trait to determine functional dominance^5^,^6^ (Table S3).

Functional diversity was evaluated by functional dispersion (FDis) (Table S3), which quantifies the mean distance of individual species to the centroid of all species in the community^50^. The weighted centroid was calculated as:


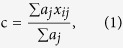


where c is the weighted centroid in the *i*-dimensional spaces, *a*_*j*_ is the abundance of species *j*, *x*_*ij*_is the attribute of species *j* for traits *i*. The FDis was calculated as:


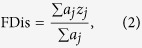


where *z*_*j*_is the distance of species *j* to the weighted centroid c. The relative abundances of species were taken into account in the calculation of FDis, which can eliminate the effects of different abundances of species in different quadrats, and can be used for individual traits and multiple traits^17^. We also analyzed the correlations between functional dominance and functional diversity of quadrats for each trait (Fig. S1) and different trait combinations (Fig. S2). Functional dominance and diversity were calculated by the “FD” package^50^ in R 3.1.1^51^.

### Aboveground C storage of tress

In this study, C storage (Mg C ha^−1^) referred to aboveground C accumulation by trees and shrubs in DHS plot. We calculated C storage for each quadrat through evaluation of the aboveground biomass of trees. Tree aboveground biomass (AGB, kg) was estimated for each individual by the allometric regression of Chave *et al*^32^. We used the model for moist forests since the annual mean precipitation was over 1500 mm (1927 mm) in DHS plot:


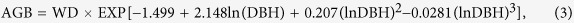


where WD is wood density in g cm^−3^, and DBH is in cm. We summed AGB of each individual for each quadrat, and C storage was calculated using following the equation^5^:


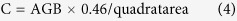


This estimation method allows us to make comparisons with many other studies using the same model^5^,^6^,^27^.

### Data analysis

Pearson correlation analysis was performed to investigate the relationships between fine-scale environmental factors, functional dominance, functional diversity, and C storage, and was also used to assess the correlations between functional dominance, functional diversity and fine-scale environmental factors. We next used the SEM to examine how fine-scale environmental factors controlled functional dominance and diversity of leaf traits (LA, LDMC and SLA), and then related to C storage. The SEMs were initiated by including all possible relationships, and the least significant relationship was then removed stepwise until all relationships were significant and the fit of the model did not increase further^37^. SEMs were conducted by the “sem” R package^52^, and we visualized the models with the R package “semPlot” (http://sachaepskamp.com/semPlot). We also used stepwise multiple regression models to evaluate the predictive ability for C storage, and compared the results from different models. In order to determine the relative importance of each variable in the final regression model, we used hierarchical partitioning method to decompose R^2^, which returned the proportion of R^2^ contributed by each regressor^53^,^54^. Hierarchical partitioning was conducted in R package “relaimpo”.

## Additional Information

**How to cite this article**: Shen, Y. *et al* Tree aboveground carbon storage correlates with environmental gradients and functional diversity in a tropical forest. *Sci. Rep.* **6**, 25304; doi: 10.1038/srep25304 (2016).

## Supplementary Material

Supplementary Information

## Figures and Tables

**Table 1:** Pearson correlation coefficients between fine-scale environmental factors, functional dominance, functional diversity and C storage in DHS plot, significant relationships are highlighted in bold.

Variable	Coefficients
Environments	
Convexity	0.34***
Soil PC1	−0.45***
Functional dominance	
LA.CWM	−0.37***
LDMC.CWM	0.49***
SLA.CWM	−0.47***
WD.CWM	0.20***
DBH.CWM	0.46***
Functional diversity	
LA.FDis	−0.38***
LDMC.FDis	0.26***
SLA.FDis	−0.37***
WD.FDis	**−**0.04
DBH.FDis	0.50***
Multi.FDis	−0.13**

Soil PC1, the first component of PCA on soil variables; LA, LDMC, SLA, WD, DBH and Multi are leaf area, leaf dry matter content, specific leaf area, wood density, maximum DBH and multivariate functional diversity, respectively; CWM and FDis indicate community weighted mean trait values and functional dispersion.

**Table 2:** Pearson correlation coefficients between fine-scale environmental factors and functional dominance, diversity in DHS plot, significant relationships are highlighted in bold.

Variable	Convexity	PC1
Functional dominance		
LA.CWM	0.38***	0.37***
LDMC.CWM	0.47***	−0.50***
SLA.CWM	−0.47***	0.49***
WD.CWM	0.38***	**−**0.03
DBH.CWM	0.09*	−0.57***
Functional diversity		
LA.FDis	−0.37***	0.43***
LDMC.FDis	−0.47***	0.30***
SLA.FDis	−0.44***	0.28***
WD.FDis	0.004	**−**0.04
DBH.FDis	0.08	−0.60***
Multi.FDis	−0.47***	**−**0.01

See Table 1 for abbreviations.

**Table 3:** Final models from multiple stepwise regressions between C storage and different component of variables (N = 500) in DHS plot.

Model		R^2^	AIC
Environment	2.43Convexity −7.73PC1 + 102.75	0.30	5014.06
Dominance	−0.27LA.CWM + 336.86LDMC.CWM + 267.75WD.CWM + 3.15DBH.CWM−294.64	0.33	4990.71
Diversity	−55.75LA.FDis −45.03LDMC.FDis + 91.10DBH.FDis + 71.44	0.34	4988.71
Environment + Dominance	1.73Convexity −4.21PC1 + 244.17WD.CWM + 2.92DBH.CWM −152.43	0.38	4959.26
Environment + Diversity	2.05Convexity −3.27PC1 −30.09LA.FDis + 71.27DBH.FDis + 48.83	0.38	4954.22
Dominance + Diversity	813.54LDMC.CWM + 0.52SLA.CWM + 329.70WD.CWM −36.36LA.FD is −27.64WD.FDis + 89.90DBH.FDis −529.93	0.39	4948.63
All variables	1.42Convexity −3.47PC1 + 724.05LDMC.CWM + 0.43SLA.CWM + 360.16WD.CWM +32.66LDMC.FDis −41.50WD.FDis + 70.54DBH.FDis −509.15	0.42	4927.25

R^2^ is adjusted coefficients for the regression model. AIC, Akaike Information Criterion. See Table 1 for abbreviations.
