# Supplementary material for: Tree aboveground carbon storage correlates with environmental gradients and functional diversity in a tropical forest
Source: Sci Rep. 2016 Jun 9;6:25304. doi: 10.1038/srep25304 (PMC4899748; doi:10.1038/srep25304)
Supplement: Supplementary Information [file srep25304-s1.pdf]

Supplementary Information

Tree aboveground carbon storage correlates with environmental gradients and functional diversity in a tropical forest

Yong Shen[1], Shixiao Yu[1], Juyu Lian[2], Hao Shen[2], Honglin Cao[2], Huanping Lu[3] & Wanhui Ye[2, *]

[1]Department of Ecology, School of Life Sciences/State Key Laboratory of Biocontrol, Sun Yat-sen University, Guangzhou 510275, PR China

[2]Key Laboratory of Vegetation Restoration and Management of Degraded Ecosystems, South China Botanical Garden, Chinese Academy of Sciences, Guangzhou 510650, PR China

[3]Ecological Meteorological Center of Guangdong Province, Guangzhou 510640, PR China

***Corresponding author:** Prof. Wanhui Ye

Email: why@scbg.ac.cn

Tel: +86 20 37252981

Table S1. Minimum, maximum, mean and coefficient of variation (CV) of topography, soil variables and plant functional traits in DHS plot.

Variable	Minimum	Maximum	Mean	CV
**Topography (N = 500)**				
Convexity (m)	-13.32	17.67	0.18	31.01
Soil (N = 500)				
Total N (mg g^{-1})	133.26	291.09	201.79	0.18
Available N (mg g^{-1})	0.47	2.76	1.18	0.43
Total P (mg g^{-1})	0.41	4.87	1.80	0.58
Available P (mg g^{-1})	0.20	0.44	0.28	0.19
Total K (mg g^{-1})	30.11	121.18	54.99	0.36
Available K (mg g^{-1})	8.45	31.22	18.15	0.19
Organic matter (mg g^{-1})	42.61	98.05	60.98	0.18
Water content (%)	10.25	27.99	18.80	0.14
pH	3.55	3.95	3.75	0.02
**Functional trait (N = 92)**				
Leaf area (cm^2)	5.98	983.42	45.45	2.34
Leaf dry matter content (g g^{-1})	0.21	0.52	0.37	0.17
Specific leaf area (cm^2 g^{-1})	63.07	266.55	139.40	0.35
Wood density (g cm^{-3})	0.28	0.85	0.58	0.20
Max DBH (cm)	1.10	90.00	24.07	0.76

Table S2. Factor loadings of the first component of principal component analysis (PCA) on soil variables in DHS plot.

Variable	PC1
Organic matter	0.372
pH	-0.359
Water content	-0.188
Total K	0.344
Available K	0.183
Total N	0.391
Available N	0.394
Total P	-0.306
Available P	0.379

Table S3. Minimum, maximum, mean and coefficient of variation (CV) of functional dominance, functional diversity and carbon storage in DHS plot.

Variable	Minimum	Maximum	Mean	CV
**Functional dominance (N = 500)**				
LA.CWM	17.43	157.31	31.97	0.52
LDMC.CWM	0.29	0.42	0.38	0.06
SLA.CWM	89.56	239.36	128.57	0.18
WD.CWM	0.49	0.72	0.63	0.05
DBH.CWM	24.92	51.86	35.18	0.15
**Functional dispersion (N = 500)**				
LA.FDis	0.04	1.21	0.16	0.98
LDMC.FDis	0.32	1.35	0.77	0.21
SLA.FDis	0.21	1.52	0.63	0.43
WD.FDis	0.23	1.00	0.66	0.21
DBH.FDis	0.27	1.43	0.82	0.25
Multi.FDis	1.02	2.40	1.74	0.14
**C storage (Mg C ha^{-1}, N = 500)**	10.80	240.73	103.19	0.42

See Table 1 for abbreviations in the text.

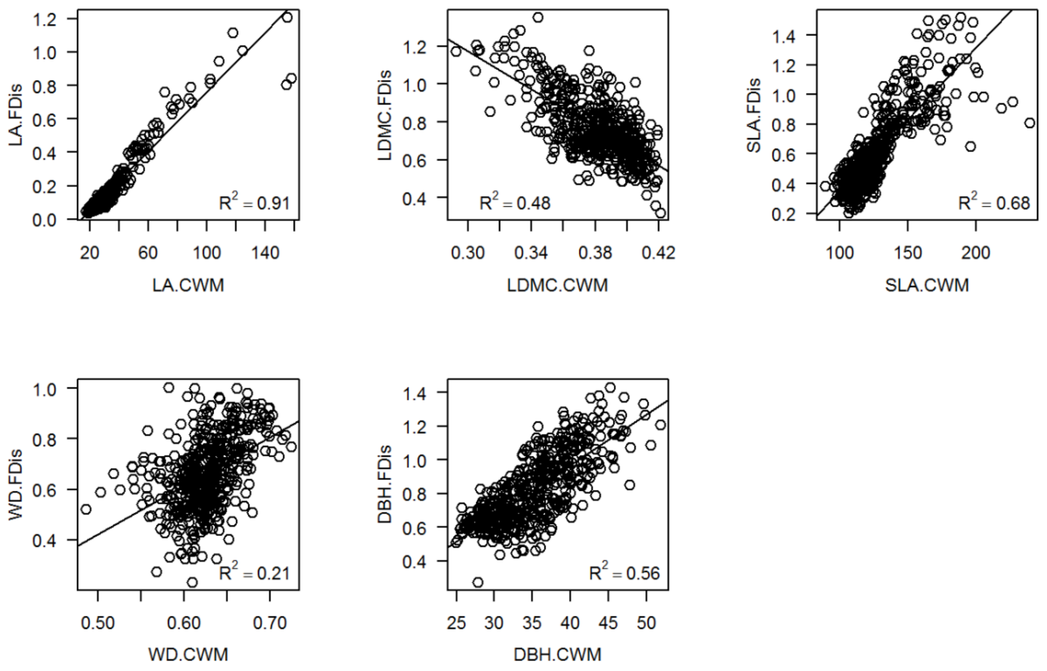

Figure S1. Functional dominance (CWM) versus functional diversity (FDis) of each trait for 500 20 m × 20 m quadrats in DHS plot. Significant relationships are shown by solid lines. See Table 1 for abbreviations.

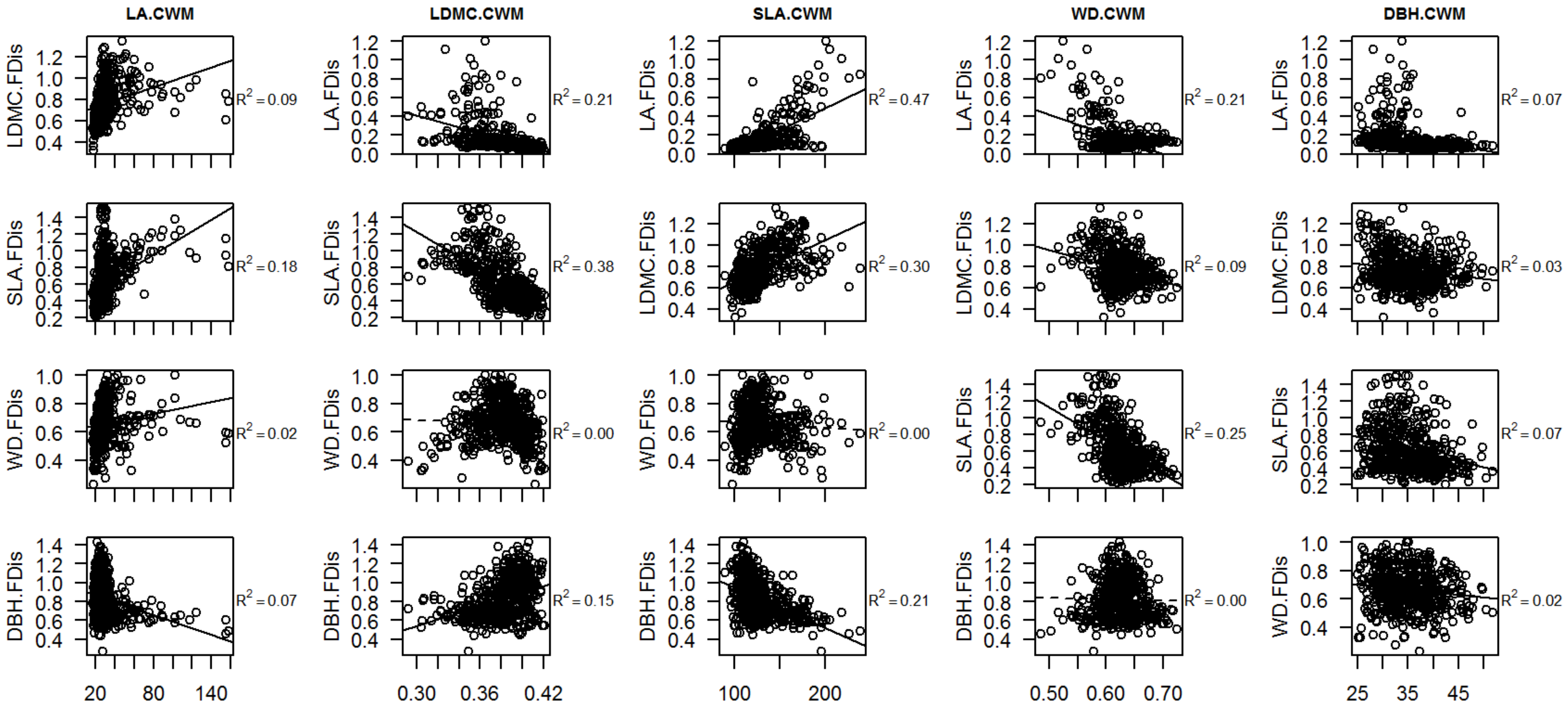

Figure S2. Functional dominance (CWM) versus functional diversity (FDis) of different traits for 500 20 m × 20 m quadrats in DHS plot. Significant relationships are shown by solid lines, and insignificant relationships are shown by dashed lines. See Table 1 for abbreviations.
